# Real world data of anticoagulant treatment in non-valvular atrial fibrillation across renal function status

**DOI:** 10.1038/s41598-022-10164-5

**Published:** 2022-04-12

**Authors:** Jose Miguel Calderon, Fernando Martinez, Antonio Fernandez, Inmaculada Sauri, Javier Diaz, Ruth Uso, Jose Luis Trillo, Josep Redon, Maria Jose Forner

**Affiliations:** 1 Cardiovascular and Renal Research Group, INCLIVA Research Institute, University of Valencia, Avda Blasco Ibañez, 17, 46010 Valencia, Spain; 2 Internal Medicine Hospital Clínico de Valencia, Valencia, Spain; 3 CIBERObn Carlos III Institute, Madrid, Spain

**Keywords:** Kidney diseases, Chronic kidney disease

## Abstract

The objective is to assess the impact of anticoagulant treatment in non-valvular atrial fibrillation (AF) and different categories of renal dysfunction in real world. Electronic Health recordings of patients with diagnosis of AF and renal function collected throughout 5 years and classified according to KDIGO categories. Stroke, transitory ischemic attack (TIA), intracranial hemorrhage and all-cause mortality were identified. Anticoagulant treatments during the study period were classified in untreated (never received therapy), VKA, NOAC and Aspirin. The risk of events was calculated by Cox-proportional hazard models adjusted by confounders. A total of 65,734 patients with AF, mean age 73.3 ± 10.49 years old and 47% females and follow-up of 3.2 years were included. KDIGO classification were: G1 33,903 (51.6%), G2 17,456 (26.6%), G3 8024 (12.2%) and G4 6351 (9.7%). There were 8592 cases of stroke and TIA, 437 intracranial hemorrhage, and 9603 all-cause deaths (incidence 36, 2 and 38 per 10^3^ person/year, respectively). 4.1% of patients with CHA2DS2-VASc Score 2 or higher did not receive anticoagulant therapy. Risk of stroke, TIA, and all-cause mortality increased from G1 to G4 groups. Anticoagulant treatments reduced the risk of events in the four categories, but NOAC seemed to offer significantly better protection. Renal dysfunction increases the risk of events in AF and anticoagulant treatments reduced the risk of stroke and all-cause mortality, although NOAC were better than VKA. Efforts should be done to reduce the variability in the use of anticoagulants even in this high risk group.

## Introduction

Atrial fibrillation (AF), the most common sustained arrhythmia in adults, is a frequent condition in aged populations increasing the risk for stroke, heart failure, dementia, and mortality^1,2^. Anticoagulant treatment^3–5^ had demonstrated a significant reduction in the risk of stroke and today is mandatory in subjects with a CHA2DS2-VASc score equal to or higher than 2^6^. Recommendations for the use of anticoagulants or antiplatelet agents in patients with CKD have been released for scientific societies^3,5^, and randomized trials have provided information about the efficacy and tolerance of treatments. Despite the evidence on the efficacy and side effects of anticoagulant treatments, their use in daily clinical practice is far from perfect due to several reasons such as lack of anticoagulant prescription despite guidelines recommendations, changes of anticoagulants during the follow-up and lack of adherence to long-term prophylactic therapies or the presence of comorbidities. Atrial Fibrillation and CKD usually coexist, and CKD not only increases the cardiovascular risk but also can alter the pharmacokinetics of the anticoagulants. Moreover, both AF and CKD share common risk factors, in fact all conditions included in the CHA2DS2-VASc score increase the risk of chronic kidney disease (CKD)^7^.

The impact of anticoagulant therapy in AF using real-world data (RWD) has been addressed in multiple studies^8–17^, but information about the scenario of AF in CKD is scarce^18^. Therefore, the study’s objective is to assess the impact of anticoagulant treatment on the risk of stroke, all-cause mortality, and major bleedings in patients with non-valvular AF and different categories of renal dysfunction.

## Subjects and methods

### Study population and baseline data collection

The sample was recruited from beneficiaries of the Valencian Health Agency’s universal health care system. The Valencian Community is a Mediterranean region located on the East-coast of Spain, with a population of 3,799,885 people older than 18 years in 2012. Every patient has a unique personal identification number for the health system, so there is one unique electronic centralized clinical record per patient. Total population data was extracted using the health information exchange function of ABUCASIS for the period of time between 1st January 2012 and 31st December 2016. ABUCASIS includes information on patient demographics, medications, vital status, past medical history and laboratory data, among others. Patients' data collected from the system during the study were documented by a process of pseudo-anonymization, making it impossible to use this information to identify the patients since the only link between the data and the patient is a code not available to the researchers. The data generated during the study was handled according the Spanish Law 3/2018 of Data Protection and Guaranty of Digital Rights the and corresponding European norms^19^. The study was reviewed and approved by the Committee for Ethics and Clinical Trials of the Hospital Clinico of Valencia. The Ethical Committee approved that the study developed under exemption of informed consent.

Forty-six thousand three hundred ninety-six subjects with diagnosis of Atrial fibrillation (ICD-9 427.31 and V07.39O; ICD-10 I48.1, I48.2, I48.91) and KDIGO stratification of risk^20^ were included in the study from 1st January 2012. Incident AF during the study period, until 31st December 2016, was also included. Information from primary care physicians, specialists, nurses’ offices, pharmacies, hospitals, and emergency departments was collected. The presence of hypertension, diabetes and dyslipidemia were collected according to previously detailed definitions^21^. CHA2DS2-VASc, HAS-BLED and KDIGO categories were calculated for each patient. Since at the beginning of the inclusion in the study not all the baseline variables were available to adjust for potential confounding, a 6-month window around the time of study inclusion were used.

### Event definitions

Incidences of stroke, transitory ischaemic attack, haemorrhagic stroke, and all-cause mortality until 31st December 2016 were collected. Events were assigned from the ICD codes recorded at discharge from hospitalizations or the emergency room. Death was extracted from the death registry. Follow-up was calculated as the difference between the starting point of taking the medication class and the date of the event, death, or 31st of December 2016, whichever occurred first.

### Renal assessment and KDIGO categories

Serum creatinine was measured and estimated glomerular filtration rate (eGFR) was calculated from creatinine, age and sex using the CKD-EPI^22^. Albuminuria and/or proteinuria was assessed in first voiding urine in the morning and expressed as the ratio with urinary creatinine (mg/g). KDIGO stratification of riskx^20^ considering eGFR and urinary albumin excretion, at baseline of the observational period study, was performed. The stratification used was the following: ***KDIGO 1*** (G1) eGFR > 60 ml/min/1.73 m^2^ and UAE < 30 mg/g creatinine; ***KDIGO 2*** (G2) eGFR > 60 ml/min/1.73 m^2^ and UAE 30–299 mg/g creatinine or eGFR 45–59 ml/min/1.73 m^2^ and UAE < 30 mg/g creatinine; ***KDIGO 3*** (G3) eGFR 30–44 ml/min/1.73 m^2^ and UAE < 30 mg/g creatinine, or eGFR 45–59 ml/min/1.73 m^2^ and UAE 30–299 mg/g creatinine, or eGFR > 60 ml/min/1.73 m^2^ and UAE > 300 mg/g creatinine; ***KDIGO 4*** (G4) eGFR 15–29 ml/min/1.73 m^2^ or eGFR 30–44 ml/min/1.73 m^2^ and UAE 30–299 mg/g creatinine or eGFR 45–59 ml/min/1.73 m^2^ and UAE > 299 mg/g creatinine.

### Anticoagulant treatment

Treatment was collected from the prescription repository of the EHR with the ATC-codes. Anticoagulant treatment was grouped in no treatment, vitamin K antagonists (VKA), B01AA (acenocumarol, warfarin), non-vitamin K antagonist Oral Anticoagulant (NOAC), B01AE (dabigatran, ribaroxaban, apixaban, edoxaban) and B01AC [aspirin (acetylsalicylic acid)]^23^. The initial treatment was the one prescribed at the time of starting the observational period or after diagnosis for incident cases, and the last treatment was the current prescription when the event occurred or at the end of the observational period. If anticoagulants were not dispensed during three months, patients were considered that were not taking treatment. For each subject, the persistence with a treatment was estimated and the time without discontinuation previous to one event or until the end of the treatment was calculated. In addition, duration of prescription stratified in those with less than 20 months, between 20 and 40 and more than 40. Subjects without any of these prescriptions during the whole follow-up period were considered untreated.

### Statistical analysis

Values are mean plus minus standard deviation and values of incidence per 1000 person/year. Incidence and survival by groups of treatment were analysed using Cox proportional hazard regression models. The risk of events based on therapy groups was evaluated by means of cumulative survival rates and Cox proportional hazards regression models. Individual time-period taking the different anticoagulants was also considered. A death is counted only toward the drug group the patient was taking at the time of death. The analysis was adjusted by potential confounders including age, sex, hypertension, diabetes, coronary heart disease, dyslipidemia, heart failure and duration of treatments. KDIGO categories were also included in some of the analysis. *Chi-square* was used to compare the incidence rates across groups of persistence with treatment. The statistical R-package was used for analysis.

## Results

### General characteristics of the study population

**Figure 1:**
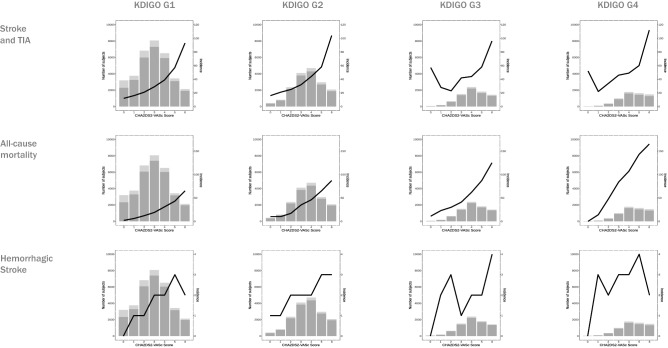
Distribution of patients by CHA2DS2-VASc Score with (dark grey) and without (light grey) anticoagulant treatment in each KDIGO category and incidence per 10^3^ patients/year of stroke and TIA (upper line), all-cause mortality (middle line) and haemorrhagic stroke (bottom line).

A total of 65,734 patients with AF, mean age 73.3 ± 10.49 years old and 47% females and follow-up of 3.2 years, were included. Hypertension was present in 87.8%, dyslipidemia in 74.7%, diabetes in 44.1% heart failure 37.4%, and coronary heart disease 27.8%. At baseline, mean CHA2DS2-VASc Score was average 3.43 (IQ 1.71–2.50) and HAS-BLED average 2.36 (IQ 1.00–2.39). According to the KDIGO classification the distribution of patients was as follows: G1 33,903 (51.6%), G2 17,456 (26.6%), G3 8024 (12.2%) and G4 6351 (9.7%). The study population’s main characteristics by the KDIGO categories are shown in Table 1 and by the ever exposure to an anticoagulant during the study in Table 2. The distribution of patients according to the CHA2DS2-VASc Score and KDIGO categories is shown in Fig. 1. The total follow-up was 2,761,922 person-months.

**Table 1:** Characteristics of the study population at baseline grouped by KDIGO category.

Variable\population	TOTAL (65,734)	KDIGO-G1 (33,903)	KDIGO-G2 (17,456)	KDIGO-G3 (8024)	KDIGO-G4 (6351)	*p* value
Age	73.31 ± 10.49	70.04 ± 10.71	75.23 ± 9.23	78.1 ± 8.41	79.46 ± 8.35	< 0.001
Sex: male	30,914 (47%)	14,512 (42.8%)	8590 (49.2%)	4370 (54.5%)	3442 (54.2%)	< 0.001
Diabetes	29,012 (44.1%)	12,833 (37.9%)	8336 (47.8%)	4261 (53.1%)	3582 (56.4%)	< 0.001
Hypertension	57,694 (87.8%)	27,974 (82.5%)	15,873 (90.9%)	7662 (95.5%)	6185 (97.4%)	< 0.001
Dyslipidemia	49,088 (74.7%)	25,116 (74.1%)	13,011 (74.5%)	6119 (76.3%)	4842 (76.2%)	< 0.001
Heart failure	24,556 (37.4%)	9344 (27.6%)	7093 (40.6%)	4187 (52.2%)	3932 (61.9%)	< 0.001
CHD	18,271 (27.8%)	8250 (24.3%)	5032 (28.8%)	2632 (32.8%)	2357 (37.1%)	< 0.001
CHA2DS2-VASc (IQ)	3.43 1.71–2.50	2.18 1.02–2.34	3.71 1.56–3.52	4.23 1.47–3.53	4.51 1.47–4.51	< 0.001
HAS-BLED (IQ)	2.36 1.00–2.39	2.18 1.02–2.32	2.44 0.95–2.34	2.56 0.89–2.34	2.85 0.95–2.32	< 0.001
NOAC	13,543 (20.6%)	7506 (22.1%)	3670 (21%)	1495 (18.6%)	872 (13.7%)	< 0.001
VKA	41,991 (63.9%)	20,589 (60.7%)	11,862 (68%)	5445 (67.9%)	4095 (64.5%)	< 0.001
Aspirin	21,707 (33%)	11,249 (33.2%)	5511 (31.6%)	2688 (33.5%)	2259 (35.6%)	< 0.001
Untreated	7244 (11%)	4166 (12.3%)	1575 (9%)	736 (9.2%)	767 (12.1%)	< 0.001

VKA vitamin K antagonists, NACO non-vitamin K antagonist oral anticoagulant, CHD coronary heart disease, IQ interquartile range.

**Table 2:** Study population grouped by anticoagulant treatment.

Variable\population	NOAC (14,588)	VKA (42,790)	ASPIRIN (22,372)	UNTREATED (6247)	*p* value
Age	72.47 ± 10.14	73.96 ± 9.24	72.84 ± 10.83	70.28 ± 14.18	< 0.001
Sex: male	6654 (45.6%)	20,595 (48.1%)	9137 (40.8%)	3047 (48.8%)	< 0.001
Diabetes	6005 (41.2%)	19,817 (46.3%)	10,676 (47.7%)	2203 (35.3%)	< 0.001
Hypertension	12,921 (88.6%)	38,473 (89.9%)	19,942 (89.1%)	4839 (77.5%)	< 0.001
Dyslipidemia	11,094 (76%)	32,643 (76.3%)	17,666 (79%)	4091 (65.5%)	< 0.001
Heart failure	5015 (34.4%)	18,436 (43.1%)	8327 (37.2%)	1316 (21.1%)	< 0.001
CHD	4042 (27.7%)	12,079 (28.2%)	9808 (43.8%)	915 (14.6%)	< 0.001
CHA2DS2-VASc (IQ)	3.29 1.70–2.42	3.57 1.62–3.51	3.40 1.76–3.50	2.95 1.94–2.44	< 0.001
HAS-BLED (IQ)	2.35 0.992.31	2.35 0.98–2.30	2.70 1.03–2.34	2.00 1.08–1.31	< 0.001
KDIGO-1	8034 (55.1%)	20,976 (49%)	11,533 (51.6%)	3678 (58.9%)	< 0.001
KDIGO-2	3960 (27.1%)	12,084 (28.2%)	5702 (25.5%)	1319 (21.1%)	< 0.001
KDIGO-3	1634 (11.2%)	5556 (13%)	2771 (12.4%)	597 (9.6%)	< 0.001
KDIGO-4	960 (6.6%)	4174 (9.8%)	2366 (10.6%)	653 (10.5%)	< 0.001

The number of subjects in the different treatments groups is superior to the number of patients since some patients received different medication in different time periods.

*VKA* vitamin K antagonists, *NACO* non-vitamin K antagonist oral anticoagulant, *CHD* coronary heart disease, *IQ* interquartile range.

### Anticoagulant treatment and events in all patients

**Figure 2:**
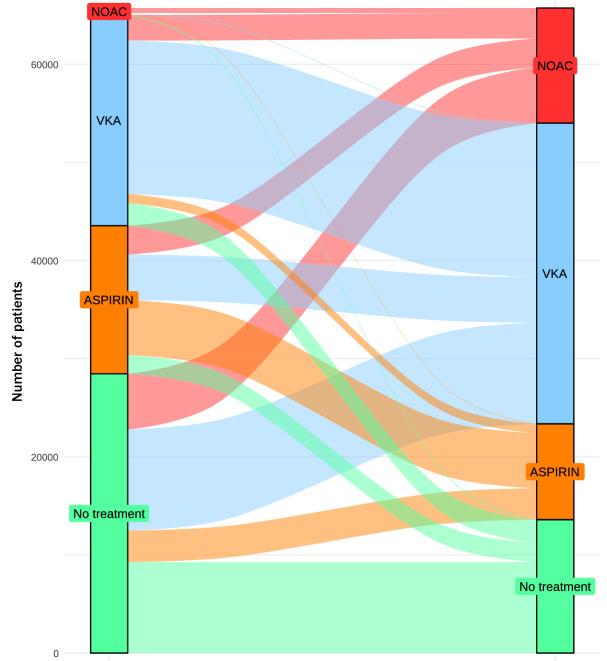
Distribution of treatments at baseline and at the end of the study period and the changes during the follow-up. Sankey diagram in Stata.

The distribution of treatments during the observational period is shown in Fig. 2. During the study period, 6247 subjects were untreated, 42,790 (65.1%) received VKA, 14,588 (22.2%), (NOAC) and 22,372 (34.0%) aspirin. The mean CHA2DS2-VASc Score was significantly lower in the untreated group, (Table 2) *p* < 0.001. Selecting those with a CHA2DS2-VASc Score equal to or greater than two, 4.1% (2626 subjects) were not receiving anticoagulant therapy in the study period, being the majority in the G1 group. The total time of treatment for each of the anticoagulant groups was 229,894 person-months in untreated subjects, 1,486,150 person-months in the VKA, 299,965 person-months in NOAC and 551,246 person-months in the aspirin group.

A total of 8592 patients suffered stroke and TIA. The overall incidence of stroke and TIA combined was 36 events/1000 patients/year. All treatments reduced the rate of incident ischemic stroke and TIA as compared to the absence of treatment, after adjusting by age, sex, CHA2DS2-VASc score, hypertension, diabetes, dyslipidemia, coronary heart disease, heart failure, KDIGO categories and time of anticoagulation, (Table 3). Those treated with NOAC had the lowest risk, followed by the aspirin group and VKA. The incidence of ischemic stroke and TIA grouped by the number of months in which the treatment was maintained is in Table 4. The higher of time under treatment was associated with lower incidence of stroke and TIA and the differences among the treated groups disappeared.

**Table 3:** Reduction of risk of each anticoagulant treatment by KDIGO category.

	Stroke and TIA	Hemorrhagic stroke	All-cause mortality
No treatment	1	1	1
**Total population**			
NOAC	0.09 (0.09, 0.10)^&^*	0.22 (0.14, 0.36)^***&***^	0.17 (0.15, 0.18)^&^*
VKA	0.22 (0.21, 0.23)^***&***^	0.71 (0.48, 1.06)	0.40 (0.37, 0.43)^***&***^
ASPIRIN	0.13 (0.12, 0.13)^&^*	0.31 (0.20, 0.48)^***&***^	0.25 (0.23, 0.27)^&^*
**KDIGO categories**			
*KDIGO G1*			
NOAC	0.08 (0.07, 0.09)^&^*^Δ^	0.14 (0.07, 0.28)^***&***^	0.14 (0.12, 0.17)^&^*^Δ^
VKA	0.20 (0.18, 0.21)^***&***^	0.53 (0.30, 0.94)	0.36 (0.31, 0.41)^***&***^
ASPIRIN	0.11 (0.10, 0.12)^&^*	0.21 (0.11, 0.39)^***&***^	0.22 (0.19, 0.26)^&^*
*KDIGO G2*			
NOAC	0.10 (0.09, 0.12)^&^*^Δ^	0.40 (0.16, 1.03)	0.18 (0.15, 0.22)^&^*
VKA	0.22 (0.20, 0.25)^***&***^	0.98 (0.42, 2.26)	0.39 (0.34, 0.45)^***&***^
ASPIRIN	0.13 (0.11, 0.15)^&^*	0.50 (0.20, 1.22)	0.22 (0.19, 0.26)^&^*
*KDIGO G3*			
NOAC	0.09 (0.08, 0.12)^&^*	0.16 (0.04, 0.60)^***&***^	0.20 (0.16, 0.25)^&^*
VKA	0.23(0.19, 0.27)^***&***^	0.62 (0.22, 1.76)	0.44 (0.37, 0.53)^***&***^
ASPIRIN	0.13 (0.11, 0.16)^&^*	0.24 (0.07, 0.78)^***&***^	0.27 (0.22, 0.33)^&^*
*KDIGO G4*			
NOAC	0.10 (0.08, 0.13)^&^*	0.27 (0.07, 1.01)	0.17 (0.14, 0.20)^&^*
VKA	0.25 (0.22, 0.30)^***&***^	0.73 (0.26, 2.08)	0.40 (0.35, 0.46)^***&***^
ASPIRIN	0.15 (0.12, 0.18)^***&***^	0.35 (0.11, 1.10)	0.27 (0.24, 0.32)^***&***^

Values are HR with the 95th confidence interval and.

*VKA* vitamin K antagonists, *NOAC* non-vitamin K antagonist oral anticoagulant.

^&^Significant differences with the untreated group.

*Significant differences with the VKA treatment.

^Δ^Significant differences with the Aspirin treatment.

**Table 4:** Incidence of ischemic stroke-TIA, haemorrhagic stroke and all-cause mortality considering the time of use of each anticoagulant treatment.

Event\incident	Incidence per 1000 patients/year			
	Persistent treatment (months)	NOAC	VKA	ASPIRIN
Stroke and TIA	< 20	344 (1164–2882) [324.23–363.77]	46 (1073–7930) [43.24–48.76]	85 (2801–13,437) [81.87–88.13]
	Between 20 and 40	49 (198–1379) [42.13–55.87]	12 (181–3767) [10.22–13.78]	39 (1513–10,817) [37.04–40.96]
	> 40	6 (84–2983) [4.76–7.24]	6 (56–1846) [4.35–7.65]	9 (788–17,737) [8.37–9.63]
Hemorrhagic stroke	< 20	8 (17–1811) [4.4–11.6]	1 (36–8039) [0.53–1.47]	3 (108–12,110) [2.38–3.62]
	Between 20 and 40	1 (5–1321) [− 0.14–2.14]	1 (19–4357) [0.51–1.49]	3 (127–11,072) [2.46–3.54]
	> 40	0 (6–3125) [0*-0.32]	0 (2–2124) [0*-0.27]	1 (121–19,602) [0.78–1.22]
All-cause mortality	< 20	128 (285–1800) [113.16–142.84]	36 (893–8084) [33.66–38.34]	61 (1991–12,085) [58.34–63.66]
	Between 20 and 40	92 (354–1320) [82.4–101.6]	19 (338–4374) [16.94–21.06]	66 (2692–11,066) [63.51–68.49]
	> 40	18 (266–3127) [15.89–20.11]	9 (89–2130) [7.2–10.8]	19 (1832–19,639) [18.13–19.87]

(Events—total) Number of patients; [95th confidence interval].

Significant differences in stroke and TIA and all cause mortality (*p* value < 0.001) for the Chi-2 for trend across categories of time use of each kind of anticoagulant treatment.

*VKA* vitamin K antagonists, *NOAC* non-vitamin K antagonist oral anticoagulant.

Intracranial hemorrhage was diagnosed in 437 subjects, incidence 2/1000 patients/years, being the risk higher in the VKA compared to those treated with NOAC and aspirin, Tables 3 and 4. A total of 2003 patients needed hospitalization due to gastrointestinal hemorrhage, 8 events/1000 patients/year, being the incidence higher in those untreated. Among those treated, the risk was higher in the VKA group.

There were 9603 deaths, incidence of all-cause mortality 38/1000 patients/year, in the study period. Treatments reduced the risk of death as compared to the absence of treatment after adjusted by potential confounders, being the lowest risk NOAC treatment followed by aspirin, Tables 3 and 4.

### Anticoagulant treatment and events by KDIGO categories

**Figure 3:**
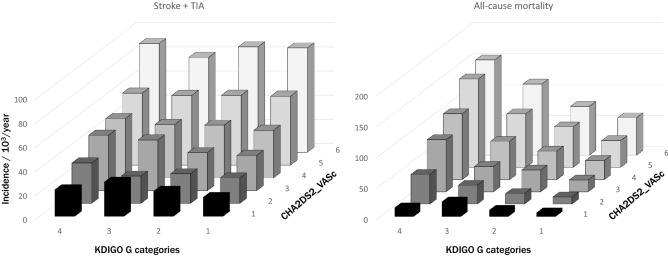
Incidence of stroke and TIA (left panel) and all-cause mortality (right panel) per 10^3^ patients/year by KDIGO categories and CHA2DS2-VASc Score.

The distribution of the anticoagulant treatments by different KDIGO categories is shown in Table 2. The proportion of untreated or treatment modality was similar in the four categories. The incidence of ischemic stroke plus TIA according the KDIGO categories and CHA2DS2-VASc Score is shown in Fig. 3 (left panel). Risk increases across the CHA2DS2-VASc Score and for each value the risk did not change for the KDIGO category. The three group of treatments, NOAC, VKA and aspirin reduce the risk in all the KDIGO groups. The reduction of risk across KDIGO categories was significantly higher for the NOAC, Table 3.

The incidence of hemorrhagic stroke, 2/1000 patients/year increased twice in the G4. Anticoagulant treatment with VKA did not reduce significantly the risk, while it was reduced by NOAC and the aspirin group, Table 3 and 4. The risk of gastrointestinal hemorrhage increased across the four KDIGO categories [HR G-2 1.41 (1.25–1.58), G-3 1.58 (1.38–1.81), G-4 2.06 (1.80–2.36)], Table 5 despite that HAS-BLED score was not significant different among the KDIGO categories (*p* < 0.05). The highest incidence was observed in patients treated with VKA.

**Table 5:** Incidence of gastrointestinal haemorrhage per 1000 patients/year in each anticoagulant treatment by KDIGO category.

Variable\population	TOTAL (46,396)	NOAC (9926)	VKA (31,519)	ASPIRIN (20,403)
KDIGO-1	4 (504–33,903) [3.66–4.34]	3 (85–7972) [2.38–3.62]	4 (355–20,956) [3.56–4.44]	3 (158–11,508) [2.47–3.53]
KDIGO-2	7 (438–17,456) [6.37–7.63]	5 (73–3911) [3.8–6.2]	7 (347–12,073) [6.23–7.77]	6 (133–5672) [4.97–7.03]
KDIGO-3	10 (283–8024) [8.89–11.11]	8 (43–1605) [5.76–10.24]	10 (213–5549) [8.66–11.34]	8 (86–2757) [6.24–9.76]
KDIGO-4	16 (334–6351) [14.33–17.67]	10 (32–938) [6.38–13.62]	16 (237–4163) [13.96–18.04]	13 (102–2341) [10.55–15.45]

(Events—total) Number of patients; [95th confidence interval].

*Significant differences (*p* value < 0.001) for the Chi-2 for trend across categories of each kind of anticoagulant treatment.

*VKA* vitamin K antagonists, *NOAC* non-vitamin K antagonist oral anticoagulant.

**Figure 4:**
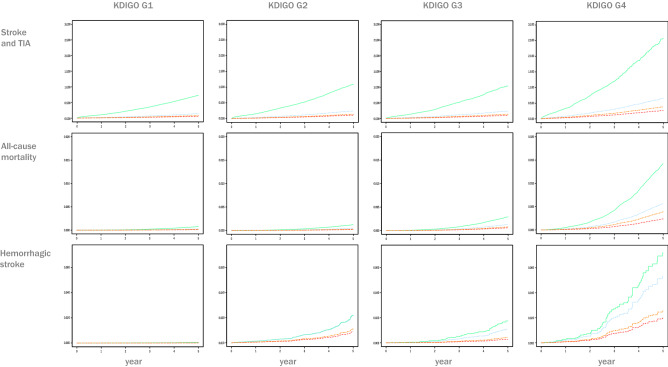
Accumulate risk of events over time in untreated and treated with VKA, NOAC or Aspirin by KDIGO categories adjusted by age, sex, CHA2DS2-VASc Score, hypertension, diabetes, dyslipidaemia, coronary heart disease, heart failure and time of treatment. Stroke and TIA (panel **A**), all-cause mortality (panel **B**) and haemorrhagic stroke (panel **C**). Hazard rate reduction of each anticoagulant treatment with the untreated as a referent are in Table 3. Lines for each treatment are Green untreated; blue VKA; Brown aspirin; Red NOAC.

In contrast with the incidence of neurologic events, all-cause mortality is dependent on both KDIGO category and CHA2DS2-VASc Score, Fig. 3 (right panel). The three groups of the anticoagulant treatment reduced the risk in the four KDIGO categories, an improvement that was less in the more advanced KDIGO categories. The NOAC offered better protection than the other treatment groups in each of the KDIGO categories, Table 3 and Fig. 4.

## Discussion

In a real-world setting of a large cohort of patients with the diagnosis of non-valvular atrial fibrillation, the renal disease stage largely influences the risk and the beneficial impact of the different anticoagulant treatment. Although all the anticoagulants reduce the risk of stroke, TIA, and all-cause mortality, the NOAC seem to offer significantly better protection than the treatment with VKA without increment in hemorrhagic events. Furthermore, significant variability in the use of anticoagulants was observed. 4.7% of patients with a CHA2DS2-VASc Score equal to or greater than 2 did not receive any anticoagulant therapy along the study period.

Randomized Clinical Trials (RCT) are the gold standard to assess drugs’ efficacy and safety, but RWD data may reflect a broader picture of clinical settings^10^. The EHR of the present study covers 92% of the population in our community and guarantees the interoperability of all sources of information. In addition, information about urinary albumin excretion was available in all subjects^16,17^. We exclude patients in KDIGO G5 or in renal replacement therapy, since the information of this group in the EHR is incomplete.

Several studies^9–12^, systematic reviews^13,14^, and meta-analysis^15,16^ have been published describing different aspects of the anticoagulation of non-valvular AF using RWD data. However, among many studies analyzing the anticoagulation in AF patients with renal dysfunction^24–38^, meta-analyses focus on RWD^34,36^ are scarce. In this increasing population, the efficacy and protection level achieve with treatments and the risk of side effects have to be considered. The meta-analysis of Dahal et al.^34^, which includes 11 primary cohorts and 2 sub studies, concluded that the use of warfarin in non-end-stage CKD resulted in a lower risk of ischemic stroke and mortality without effect on major bleedings. The beneficial impact of treatment was not present for patients in end-stage kidney disease (ESKD). Ding et al.^36^, compared a register, the Murcia AF Project, with the AMADEUS clinical trial in terms of the impact of VKAs in the incidence of ischemic stroke, major bleeding, all-cause mortality, myocardial infarction, and intracranial hemorrhage in subjects with eGFR < 60 ml/min/1.73 m^2^. The annual risk of ischemic stroke, major bleeding and all-cause mortality was significantly higher, more than twice, in the register compared with those in the clinical trial. The authors conclude that risk may be under-estimated in the environment of randomized control trials. Another registry^37^ concludes that the incidence rates of stroke, mortality, and bleeding increase with reducing eGFR across the whole range of renal function. In this study, anticoagulant treatment reduced stroke and mortality risk at one year compared with untreated patients. Likewise, in a Nationwide Observational cohort study^38^, non-ESKD was associated with a higher risk of stroke/thromboembolism across risk strata in AF patients. Those with high-risk, CHA2DS2-VASc Score equal to or higher than two, benefited from warfarin treatment for stroke prevention. In contrast patients in ESKD, treatment with warfarin increases the risk of hemorrhagic stroke^39^.

The present study expands the information available on the renal function’s impact on the risk of AF complications in real world. We have observed that: (i) incidence of neurological events, stroke and TIA, increases across the CHA2DS2-VASc Score and for each value the risk did not change for the KDIGO categories; (ii) anticoagulant treatment reduces the risk of neurological events and mortality across the spectrum of renal dysfunction in subjects with GFR > 15 ml/min/1.73 m^2^; (iii) NOAC were superior to VKA across the KDIGO categories, while superiority over aspirin was present in the lowest renal damage categories, KDIGO 1 and 2; (iv) aspirin also reduced risk of stroke and TIA and mortality across the renal dysfunction categories, as well as reduced risk of hemorrhagic stroke in the lowest renal damage categories, KDIGO 1–3 and (v) hemorrhagic events, neurologic and in the gastrointestinal tract were lower in the NOAC group. Results concerning gastrointestinal hemorrhage may have selection bias due to the choice of treatment was influenced by the bleeding risk of patients. The fact that the gastrointestinal hemorrhage was higher in the untreated subjects is probably because many of these patients had high bleeding risk and therefore were not treated with anticoagulants.

Several studies have compared the NOAC and VKA in terms of stroke risk reduction or major bleeding events with better results for NOAC in some studies^26,32^ but no others^33^. In the present study performed in patients with KDIGO 1–4 and in the absence of ESKD we confirm the superiority of NOAC. Superiority of NOAC in reduction of stroke over to VKA disappeared in patients with a very low GFR or in dialysis, although experienced fewer bleeding events with NOAC^40,41^. In fact, pivotal RCT have demonstrated a net clinical benefit for NOACs versus VKA with mild-moderate CKD, but there is little evidence in patients with AF and stages 4^42^. Further benefit of NOAC over VKA is in the incidence of anticoagulant-related nephropathy, mainly described after the introduction of VKA treatment although some case with the use of NOAC have been reported^43^. Finally, it is worthy to comment the fact that the difficulty to achieve anticoagulation in range for the VKA is greater when lowest is the GFR. In the present study, the relatively low use of NOAC depends on the study period, 2012–2016, and prescription limitations within the EHR due to their cost.

Less attention had been paid to the treatments with aspirin. What is relevant of the present data is the fact that aspirin also reduce the risk of stroke and TIA and mortality without increasing the risk of intracranial bleeding in the present study in KDIGO 1–3 but not in KDIGO 4. Protection it was inferior to NOAC but not to VKA. Whether or not the benefit of aspirin is due to a selection bias at the time of inclusion needs to be considered. Despite this observation, patients with a CHA2DS2-VASc Score 2 or higher needs anticoagulant treatment^44^.

Lack of persistence is a relevant point when RWD are analyzed. One study observed suboptimal persistence to NOAC in patients with AF, with 1 in 3 patients adhering to their NOAC < 80% of the time and the lack of persistence was associated with poor clinical outcomes^13^. In the present study, it is worthy to comment, the longer the duration of anticoagulant treatment largely reduced the risk of stroke and mortality. The inclusion of RWD in meta-analyses could help evaluate the effectiveness of health care interventions^15^ and RW cost-effectiveness studies could assist policy-makers for an optimal allocation of resources^45^.

Strengths and limitations of the study should be contemplated. A large number of patients with AF was analyzed with a long follow-up accounting for potential confounders such age, sex, and major cardiovascular risk factors. Moreover, the time of drug prescription was considered. Some limitations such as the presence of a high percentage of missing values are inherent to the EHR. To minimize its impact, only patients with the required variables for the analysis were selected. Renal parameters, eGFR and albuminuria were assessed at the time of starting the study or when the incident AF occurs, then it is possible that during the study some changes in the renal status have been produced. The reasons for the lack of treatment, the quality of VKA control, and the dosage and type of NOAC were not analyzed. Challenging patients with End Stage Renal Disease were excluded. Finally, even that the time period of the study was in which NOAC were progressively introduced, there is a large number of patients treated with this drug class.

The 2020 ESC guidelines on diagnosis and management of AF had upgraded treatment recommendations for switching from VKAs to NOAC therapy^5^. It is recommended or indicated for patients on VKA who have a time in the therapeutic range below 70%, mainly in subjects with reduced eGFR. According to the present data, anticoagulant treatment reduced stroke and TIA risk, as well mortality, in patients with KDIGO stage 4 including patients with GFR > 15 ml/1.73 m^2^, then NOAC treatment could be recommended but prospective control studies are required, but renal function should be monitored. Finally, a major goal is to increase the percentage of treated patients and their adherence to the treatment.

In conclusion, the present study demonstrated that the use of anticoagulant treatment in real life is far from guidelines’ recommendations. A high proportion of high-risk untreated subjects was detected. Although potential advantages of NOAC compared to VKA were present across different degrees of renal dysfunction, we must increase the prescription rate to reduce mortality and stroke incidence.
